# Retraction Note: Overexpression of A613T and G462T variants of DNA polymerase β weakens chemotherapy sensitivity in esophageal cancer cell lines

**DOI:** 10.1186/s12935-019-0814-1

**Published:** 2019-04-08

**Authors:** Yuanyuan Wang, Xiaonan Chen, Qianqian Sun, Wenqiao Zang, Min Li, Ziming Dong, Guoqiang Zhao

**Affiliations:** 1 School of Basic Medical Sciences, Zhengzhou University, No.100 Kexue Road, 450001 Zhengzhou, Henan China; 2 Collaborative Innovation Center of Cancer Chemoprevention of Henan, 450001 Zhengzhou, Henan China

## Retraction to: Cancer Cell Int (2016) 16:85 10.1186/s12935-016-0362-x

The Editor-in-Chief has retracted this article [1] because Figure 6a and 6b show overlap with Figure 4b in [2]. An investigation by Zhengzhou University has confirmed that these figures overlap. The data reported in this article are therefore unreliable.

Guoqiang Zhao agrees with this retraction. Yuanyuan Wang, Xiaonan Chen, Qianqian Sun, Wenqiao Zang, Min Li, and Ziming Dong have not responded to any correspondence about this retraction.
